# An Empirical Model Linking Physico-Chemical Biomaterial Characteristics to Intra-Oral Bone Formation

**DOI:** 10.3390/jfb14070388

**Published:** 2023-07-22

**Authors:** Ehsan Sadeghian Dehkord, Greet Kerckhofs, Philippe Compère, France Lambert, Liesbet Geris

**Affiliations:** 1 GIGA In Silico Medicine, Biomechanics Research Unit (Biomech), University of Liège, 4000 Liège, Belgium; ehsan.sadeghian@kuleuven.be; 2 Prometheus, Division for Skeletal Tissue Engineering, Katholieke Universiteit Leuven, 3000 Leuven, Belgium; greet.kerckhofs@uclouvain.be; 3 Biomechanics Laboratory, Institute of Mechanics, Materials, and Civil Engineering (iMMC), Université Catholique Louvain, 1348 Louvain-la-Neuve, Belgium; 4 Institute of Experimental and Clinical Research (IREC), Université Catholique Louvain, 1200 Woluwé-Saint-Lambert, Belgium; 5 Department of Materials Engineering (MTM), Katholieke Universiteit Leuven, 3000 Leuven, Belgium; 6 Laboratory of Functional and Evolutionary Morphology, FOCUS Research Unit, Department of Biology, Ecology and Evolution, University of Liège, 4000 Liège, Belgium; pcompere@uliege.be; 7 Center for Applied Research and Education in Microscopy (CAREM) and Biomaterials Interfaculty Center (CEIB), University of Liège, 4000 Liège, Belgium; 8 Department of Periodontology, Oral Surgery and Implant Surgery, Faculty of Medicine, University Hospital of Liège, 4000 Liège, Belgium; france.lambert@chuliege.be; 9 Dental Biomaterials Research Unit (d-BRU), University of Liège, 4000 Liège, Belgium; 10 Department of Mechanical Engineering, Division of Biomechanics (BMe), Katholieke Universiteit Leuven, 3000 Leuven, Belgium

**Keywords:** empirical modeling, intra-oral bone formation, calcium phosphate, physico-chemical, biomaterials

## Abstract

Facial trauma, bone resection due to cancer, periodontal diseases, and bone atrophy following tooth extraction often leads to alveolar bone defects that require bone regeneration in order to restore dental function. Guided bone regeneration using synthetic biomaterials has been suggested as an alternative approach to autologous bone grafts. The efficiency of bone substitute materials seems to be influenced by their physico-chemical characteristics; however, the debate is still ongoing on what constitutes optimal biomaterial characteristics. The purpose of this study was to develop an empirical model allowing the assessment of the bone regeneration potential of new biomaterials on the basis of their physico-chemical characteristics, potentially giving directions for the design of a new generation of dental biomaterials. A quantitative data set was built composed of physico-chemical characteristics of seven commercially available intra-oral bone biomaterials and their *in vivo* response. This empirical model allowed the identification of the construct parameters driving optimized bone formation. The presented model provides a better understanding of the influence of driving biomaterial properties in the bone healing process and can be used as a tool to design bone biomaterials with a more controlled and custom-made composition and structure, thereby facilitating and improving the clinical translation.

## 1. Introduction

Guided bone regeneration (GBR) is a therapeutic strategy pursued in dental sciences for its potential to treat periodontal and maxillofacial defects and bone atrophies following tooth extraction. Bone substitute biomaterials that support alveolar augmentation play a key role in ensuring the success of the bone regeneration process [1]. Although autologous bone grafts have long been (and still are) considered a gold standard for their osteoconductivity, osteogenicity, and structure [2,3], they have potentially substantial disadvantages restricting their applications such as the limited bone volume available for harvesting, morbidity, and discomfort at the donor site and the difficulty of getting the form into desirable shapes [4,5]. Hence, guided bone regeneration using allogenic, xenogenic, or synthetic biomaterials has been suggested as an alternative approach to autologous bone grafts [6].

An ideal bone graft in the dental field is expected to serve as an integrated and (very) slowly biodegradable 3D environment that properly exhibits: (i) biocompatibility, (ii) osteoconductivity to guide bone tissue formation, and ideally (iii) osteoinductivity to stimulate and activate host osteoprogenitor cells from surrounding tissues [7,8,9]. These outcomes are influenced by the physico-chemical properties of the biomaterial such as interconnected porosity, mechanical integrity, chemical composition, surface topology, and dissolution behavior [10,11]. As these properties are mostly coupled, the debate is still ongoing on what constitutes the optimal biomaterial characteristics for a particular clinical application [8,12,13].

Of the aforementioned physico-chemical characteristics, surface roughness has been described as a determining factor for the host response in intra-oral biomaterials [14]. The influence of a biomaterial’s surface roughness is a multi-faceted topic in which the topography, the chemistry, and the physics of the surface have attracted the most attention from researchers and manufacturers of alveolar bone biomaterials. The surface composition, purity, and roughness of the biomaterial seem to be critical to early successful material and tissue interactions affecting osteointegration and bone formation [15]. Although smooth surfaces are favorable in soft tissue engineering, both in terms of cell anchoring and growth [16], rougher surfaces have better outcomes in terms of bone deposition [15]. Chemical composition is another critical factor in the osteoinductivity of biomaterials and greatly affects vascularization as it directly interacts with endothelial cells during vessel formation. In particular, for bioceramics, the chemical composition determines the bioactivity and degradation rate of biomaterials [17,18,19,20]. Macroporosity is also described as a key factor in the alveolar bone grafts and refers to the presence of macro-pores, being pores with diameters above 100 µm. Macroporosity is known to facilitate osteogenesis and angiogenesis [21]. The existence of interconnected macropores in the bone biomaterials is critical during the early stage of tissue ingrowth on porous scaffolds and provides better body fluid circulation and cell migration to the core of the implant [22,23]. Besides the aforementioned properties, other physico-chemical factors such as microporosity and mechanical stability have been regarded to influence the *in vivo* performance of bone biomaterials [24,25].

Several studies have focused on examining the combined impact of biomaterial properties on the resulting bone formation using data-driven methods [26,27,28,29]. These investigations have developed empirical models to quantitatively assess the influence of biomaterial characteristics on *in vivo* (ectopic) bone formation, though without specifically addressing maxillofacial bone regeneration, which is the primary objective of our study. For example, the bone-forming capacity of cell-seeded CaP scaffolds has been correlated with the pore shape, chemical composition, and the amount of seeded cells in one study [26], and surface area, grain size, and volume fraction in another study [27]. These studies have primarily concentrated on the influencing factors of cell-seeded scaffolds in tissue regeneration and have not specifically investigated the effects of biomaterial characteristics alone. In addition, certain systematic reviews and meta-analyses investigated the clinical outcomes of biomaterials for alveolar bone regeneration, however, the effect of the structural characteristics of the bone substitutes on those outcomes was poorly investigated [30,31,32]. As such, a comprehensive view of the influence of multiple physico-chemical factors in intra-oral bone regeneration has not yet been reported.

**Figure 1:**
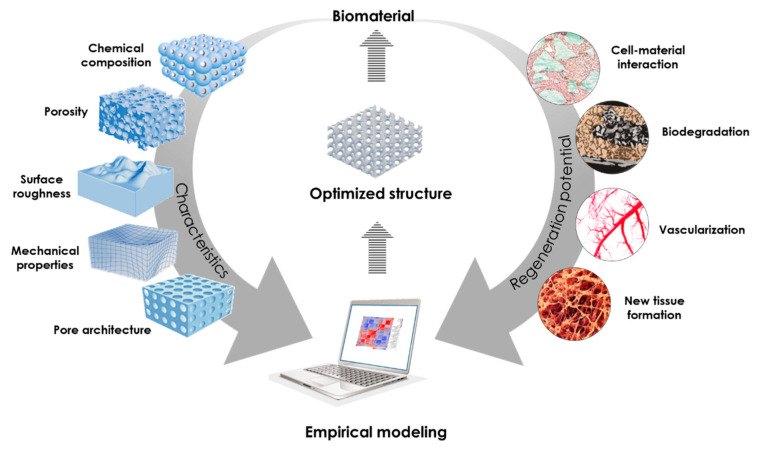
Illustration of the empirical modeling strategy used in this study to link the biomaterial’s physico-chemical characteristics (left arrow) and the regeneration potential (right arrow).

In view of this, the purpose of this study is to develop an empirical model linking intra-oral bone regeneration to the biomaterial’s physico-chemical characteristics. By examining the distinct topographical and compositional properties of various biomaterials, we aim to assess their impact on the regenerative potential of bone biomaterials. Hereto, a quantitative data set is built composed of physico-chemical characteristics of commercially available intra-oral bone biomaterials, including topography, chemical composition, porosity and surface roughness, and their *in vivo* response when implanted in a sinus augmentation animal model. The empirical model based on the aforementioned data aims to provide a tool to better understand the (combined) influence of driving biomaterial properties on the *in vivo* bone regeneration response as well as to design bone biomaterials with more controlled and custom-made structures (Figure 1).

## 2. Materials and Methods

### 2.1. Graft Types

Seven different types of commercially available alveolar grafts have been evaluated in this study. They include BioOss^®^ (Geistlich Pharma AG, Wolhusen, Switzerland), BioOss^®^-Collagen (Geistlich Pharma AG, Wolhusen, Switzerland), MP3^®^ (Osteobiol, Torino, Italy), Ostim^®^ (Heraeus Kulzer GmbH, Hanau, Germany), Cerasorb^®^ (Curasan AG, Germany), BoneCeramic^®^ (Straumann, Switzerland), and Natix^®^ (Tigran Technologies AB, Malmo, Sweden). The first four of these biomaterials are composed of hydroxyapatite (HAp) particles. BioOss^®^ is the mineral component of bovine bone. BioOss^®^-Collagen is granules of BioOss^®^ mixed with 10% highly purified porcine collagen. MP3^®^ consists of 90% cortico-cancellous porcine bone and 10% collagen. Ostim^®^ is nanocrystalline precipitated HAp with a viscous and paste-like consistency. Cerasorb^®^ is made of pure-phase β-tricalcium phosphate (β-TCP) in granular form for dental application. BoneCeramic^®^ is a synthetic bone substitute of medical-grade purity that is composed of biphasic calcium phosphate (BCP; the mixed combination of HAp and β-TCP). Natix^®^ is the only biomaterial that is not CaP-based in this study. It is composed of porous granules in irregular shapes made of commercial pure grade-one titanium (Ti). The chemical composition and characteristics of these biomaterials are summarized in Table 1.

### 2.2. In Vivo Experiment

In the previous in-house studies [33,34,35], the 7 biomaterials described in Table 1 were implanted in bilateral sinus-lift procedures in rabbits. Those studies were part of a major project including 96 sinus-lift procedures performed on 48 New Zealand white rabbits (adult, males, average body weight of 3.0 kg) using 10 different types of bone grafts assessed at three distinct time points: 1 week, 5 weeks, and 6 months. Specifically, the biomaterials were randomly allocated to the sinuses and 16 rabbits were sacrificed at each time point, so that at least three sinuses were available for each graft at each time point, yielding a two-factor experimental design (graft and time) with repeated measurements. All experimental procedures and protocols were reviewed and approved by the Institutional Animal Care and Use Ethics Committee of the University of Liège (ethical file number: 583), Belgium, and fully described in the corresponding studies [33,34,35]. Animals were sacrificed at each time point and samples were dissected, fixed for a week, and prepared for histomorphometrical assessment quantifying bone-to-material contact (BMC) as well as bone density, and regenerated area. Using SEM, the regions of interest (ROI) were manually defined, and the following areas were automatically calculated:

- Regenerated area was defined as the percentage of raw surface colonized by newly formed bone per total zonal area (*n* = 6).
- BMC was measured as the percentage of particles perimeter in contact with the newly formed bone.

Histological findings have been analyzed in those published works [33,34,35]. In this study, regenerated area and BMC at 6 months were used as the measure for *in vivo* outcomes (*n* = 6). Moreover, the surface invaded by cell colonization was measured for different time points, which was further used to calculate the macroporosity of the biomaterials.

### 2.3. Characterization of Explanted Grafts

In the context of this study, additional analyses were performed on samples collected from the abovementioned studies. All the collected samples from the previous studies were fixed in ethanol and embedded in polymethylmethacrylate (PMMA) resin. Due to the absence of raw materials, samples explanted after 1 week *in vivo* were used as surrogates to determine the surface roughness of the original materials.

#### 2.3.1. ESEM Observation

The samples were sectioned and mirror-polished to be observed in an Environmental Scanning Electron Microscope (ESEM, FEI ESEM-XL 30) working in a low-vacuum condition of 0.4 Torr (with water vapor as gas in the chamber) to avoid metal coating. Images were acquired at different magnifications with the large-field gaseous secondary electron detector (GSED) to see the section surface morphology through secondary electrons and with the backscattered electron detector (BSE-detector) to reveal the sectioned graft biomaterials and bone trabeculae through the high Z-contrast between the minerals and the resin-embedded soft tissues. The observation conditions were 15 kV of accelerating voltage, spot size 3.0, and 10 mm of working distance as indicated in the black mask of each with magnification and detector used [36,37].

#### 2.3.2. Surface Roughness Analysis

One main difficulty in evaluating the roughness of bone substitutes in the form of granules is the high waviness of the material due to their shape and porosity. This limits the use of conventional methods such as contact-based profilometry or laser profilometry in acquiring their surface roughness. In this study, we used ESEM along with an in-house MATLAB^®^ tool to acquire surface profiles of the bone grafts for surface roughness evaluation [38]. This MATLAB^®^ tool that has been specifically developed for quantification of surface roughness allows non-destructive assessment of the micro-scale roughness of porous materials at their outer surface as well as inside the structures when combined with µCT imaging. For each type of biomaterial, three images at 2000× magnification obtained from ESEM images of the explants were analyzed by defining a minimum evaluation length of 40 µm over the biomaterial’s surface, and the following surface roughness parameters were measured:

- Arithmetical mean deviation of surface roughness

(1) $$P_{a}=\frac{1}{n}\;\sum_{i=1}^{n}\left|y_{i}\right|$$

- Root-mean-square deviation of surface topography

(2) $$P_{q}=\sqrt{\frac{1}{n}\;\sum_{i=1}^{n}{y_{i}}^{2}}$$

- Total height of the roughness profile

(3) $$P_{t}=P_{p}-P_{v}$$

where *n* is the number of data points in the X-direction, *y* is the surface height relative to the mean plane, $P_{p}$ is the maximum profile peak height, and $P_{v}$ is the maximum profile valley depth.

#### 2.3.3. Macroporosity Measurement

Based on SEM observation of the bone grafts explanted in rabbit *in vivo* studies [33,34,35], the macroporosity of the scaffold, including the interparticle voids, was calculated as the percentage of the surface invaded by cell colonization per total zonal area (area of soft tissue and marrow spaces/total zonal area). For each type of biomaterial, the values of the first week (6 samples for each graft) were analyzed to calculate the average macroporosity for a given biomaterial.

### 2.4. Empirical Model

A quantitative data set was built composed of physico-chemical characteristics of the biomaterials and their *in vivo* response. The morphological properties included chemical composition (as mentioned in Table 1) as well as macroporosity and surface roughness (defined as described in previous sections). Partial least square regression (PLSR) modeling was applied to the data set in order to find out which (combination of) physico-chemical characteristics would allow predicting the bone regenerative response after 6 months of *in vivo* implantation, as quantified by the BMC measured from histomorphometry. PLSR is able to identify the information content within the set of measured physico-chemical characteristics that most closely map onto the output response (amount of BMC). The resulting mapping of lumped signals to corresponding responses then allows identifying the most “important variables” for the *in vivo* bone formation within the investigated set of biomaterial characteristics. This mathematical formalism has previously been shown to be capable of relating quantitative contributions of multiple signals to a (single) measured response [28,39,40]. A leave-one-out strategy was employed to construct a cross-validation model, avoiding overfitting and assessing the potential of the empirical model to be applied to other new materials not present in the training data set. The PLSR analysis was performed using JMP Pro software, v11 (Sas, NC, USA).

### 2.5. Statistical Analysis

All data from quantitative experiments including characterization methods and also *in vivo* data were statistically analyzed. The data are expressed as mean ± standard deviation (SD). To compare multiple groups’ means with two or more parameters, statistical analysis of the results was performed by one-way analysis of variance (ANOVA) followed by post hoc tests (Tukey’s multiple comparison test). All the graphs, calculations, and statistical analyses were performed using GraphPad Prism software version 8.2.1 for Windows (GraphPad Software, San Diego, CA, USA). In all graphs, significances are indicated as follows: * *p* < 0.05, ** *p* < 0.01, *** *p* < 0.001, and **** *p* < 0.0001.

## 3. Results

### 3.1. In vivo Regeneration

**Figure 2:**
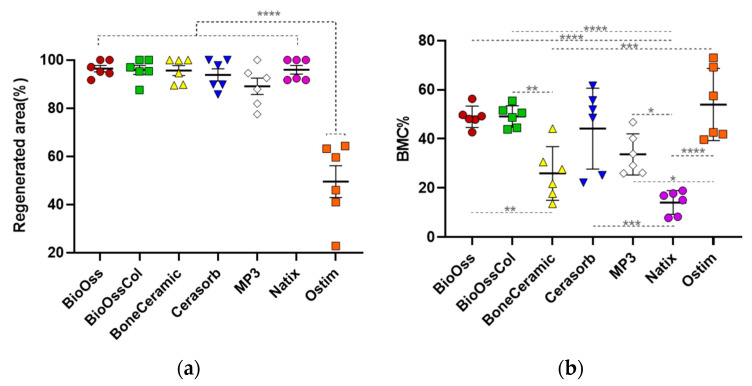
The calculated amounts of (**a**) regenerated area and (**b**) BMC for the biomaterials are shown as mean ± SD (red: BioOss^®^, green: BioOss^®^-Collagen, yellow: BoneCeramic^®^, blue: Cerasorb^®^, white: MP3^®^, purple: Natix^®^ and orange: Ostim^®^). One-way ANOVA test; * *p* < 0.05, ** *p* < 0.01, *** *p* < 0.001 and **** *p* < 0.0001.

Within the histomorphometrical assessment in the previous in-house studies, the ROIs were defined manually for all explanted samples, and the different areas of newly formed bone, bone graft, and noncalcified tissue were calculated automatically [33,34,35]. The histomorphometrical data was available for three time points (1 week, 5 weeks, and 6 months). To predict the bone forming capacity of bone grafts, the values of the regenerated area and BMC at the longest period (6 months) were used in this study. The regenerated area was calculated as the percentage of raw surface invaded by new bone per total defect surface [33,34,35] (Figure 2a). BioOss^®^ showed the highest percentage of regeneration area at 96.42 ± 3.27% and Ostim^®^ the lowest area at 49.47 ± 16.14%, which was significantly lower than other bone grafts. The regenerated area was 95.95 ± 4.44% in Natix^®^ and 95.87 ± 4.57% in BioOss^®^-Collagen, rather similar to BioOss^®^. Calculations showed a relatively high percentage of area for BoneCeramic^®^, Cerasorb^®^ and MP3^®^ with 95.63 ± 5.09%, 93.83 ± 6.17% and 85.02 ± 15.43%, respectively.

The bone-to-material contact (BMC) was defined as the percentage of particle perimeter in contact with the newly formed bone (Figure 2b). Despite the low level of bone regeneration, Ostim^®^ showed the highest amount of BMC with 53.98 ± 14.7%. It makes sense as the nanoparticles of Ostim^®^ are expected to provide more surface for new bone tissue formation. Natix^®^ displayed the lowest amount of BMC at 14.12 ± 4.8%; however, it had a larger regeneration area. Both BioOss^®^ grafts were in the same range of BMC at 49.01 ± 4.4% and 48.99 ± 4.3% for the ones without and with collagen, respectively. The calculated amount of BMC for other grafts was 44.17 ± 16.5% for Cerasorb^®^, 33.68 ± 8.3% for MP3^®^ and 25.94 ± 10.9% for BoneCeramic^®^.

### 3.2. Graft Characterization

#### 3.2.1. Surface Roughness Analysis

**Figure 3:**
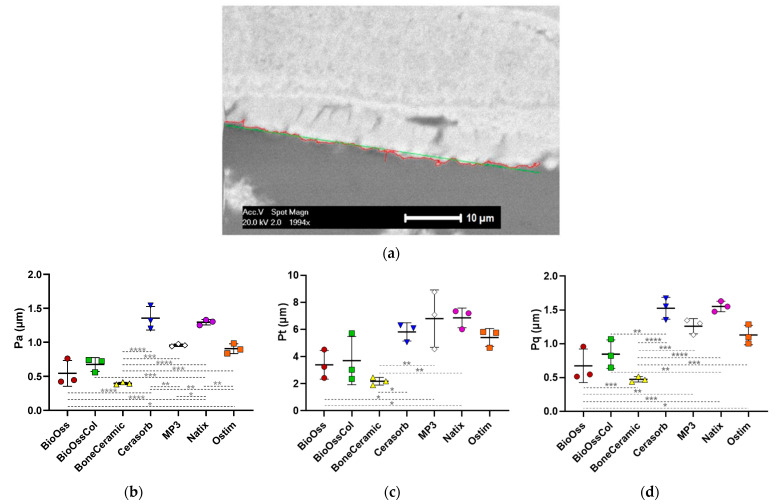
(**a**) Surface roughness measurement of a representative BioOss^®^ sample using the in-house developed MATLAB^®^ tool [26] based on the profile lines of the sample surface in the binarized ESEM image. (**b**–**d**) The values of P_a_, P_t_, and P_q_ for the biomaterials are shown as mean ± SD (red: BioOss^®^, green: BioOss^®^-Collagen, yellow: BoneCeramic^®^, blue: Cerasorb^®^, white: MP3^®^, purple: Natix^®^ and orange: Ostim^®^). One-way ANOVA test; * *p* < 0.05, ** *p* < 0.01, *** *p* < 0.001 and **** *p* < 0.0001.

The micro-scale surface roughness of the samples was analyzed and the surface roughness parameters including P_a_, P_t_ and P_q_ were quantified (Figure 3a). Roughness values in Natix^®^; the only non-CaP graft, were relatively highest amongst all biomaterials (P_a_ = 1.29 ± 0.04 µm, P_t_ = 6.85 ± 0.72 µm and P_q_ = 1.55 ± 0.07 µm), except for P_a_ which was observed highest in Cerasorb^®^ (P_a_ = 1.35 ± 0.17 µm). The other roughness values for Cerasorb^®^ were P_t_ = 5.81 ± 0.66 µm and P_q_ = 1.52 ± 0.16 µm. After Natix^®^ and Cerasorb^®^, MP3^®^ was in the top range of roughness values with P_a_ = 0.95 ± 0.02 µm, P_t_ = 6.80 ± 2.12 µm and P_q_ = 1.26 ± 0.11 µm.

The analysis indicated the same order of values for other bone grafts. Ostim^®^ showed parameters of P_a_ = 0.90 ± 0.07 µm, P_t_ = 5.40 ± 0.65 µm and P_q_ = 1.13 ± 0.14 µm. Of note, the Ostim^®^ nanoparticles are expected to be clustered in the defect site and the corresponding roughness values are the roughness of clumped surface formed by nanoparticles. BioOss^®^-Collagen (P_a_ = 0.67 ± 0.10 µm, P_t_ = 3.70 ± 1.77 µm and P_q_ = 0.84 ± 0.20 µm) and BioOss^®^ (P_a_ = 0.54 ± 0.19 µm, P_t_ = 3.40 ± 1.04 µm and P_q_ = 0.67 ± 0.24 µm) had the similar roughness values which make sense as both have the same range of particle sizes, due to their production by the same fabrication. In the end, roughness analysis showed the lowest values for BoneCeramic^®^ with P_a_ = 0.39 ± 0.01 µm, P_t_ = 2.18 ± 0.28 µm and P_q_ = 0.47 ± 0.04 µm. The roughness values for different biomaterials are shown in Figure 3b–d.

#### 3.2.2. Macroporosity Measurement

**Figure 4:**
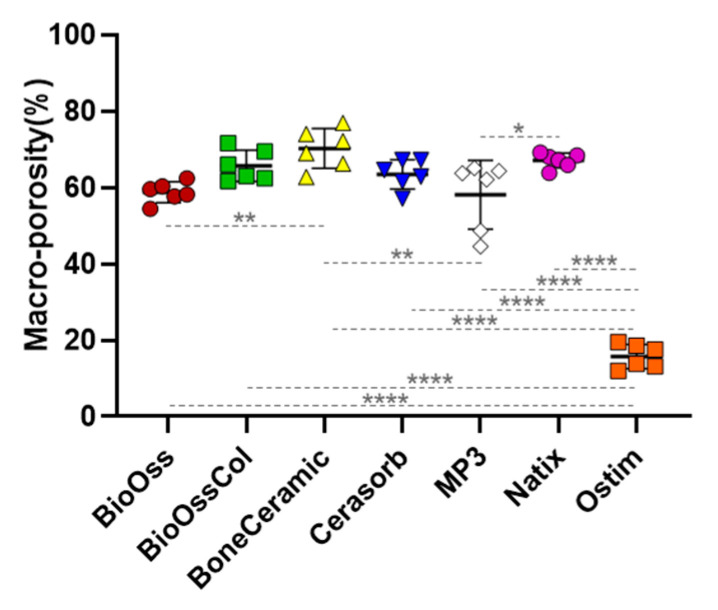
The values of macroporosity for all the bone grafts are shown as mean ± SD (red: BioOss^®^, green: BioOss^®^-Collagen, yellow: BoneCeramic^®^, blue: Cerasorb^®^, white: MP3^®^, purple: Natix^®^ and orange: Ostim^®^). One-way ANOVA test; * *p* < 0.05, ** *p* < 0.01 and **** *p* < 0.0001.

In the macroporosity analysis (Figure 4), BoneCeramic^®^ showed the highest value at 70.23 ± 5.21%. Ostim^®^ (15.81 ± 3.18%) was significantly lower than other biomaterials. This can be attributed to its nanostructure which rarely provides pores above 100 µm.

Natix^®^, BioOss^®^-Collagen, and Cerasorb^®^ showed the same range of macroporosity at 67.12 ± 1.94%, 65.73 ± 4.07%, and 63.44 ± 3.83%, respectively, while BioOss^®^ and MP3^®^ had the close value of macroporosity to each other at 58.79 ± 2.72% and 58.12 ± 9.02% in order.

### 3.3. Empirical Model

**Figure 5:**
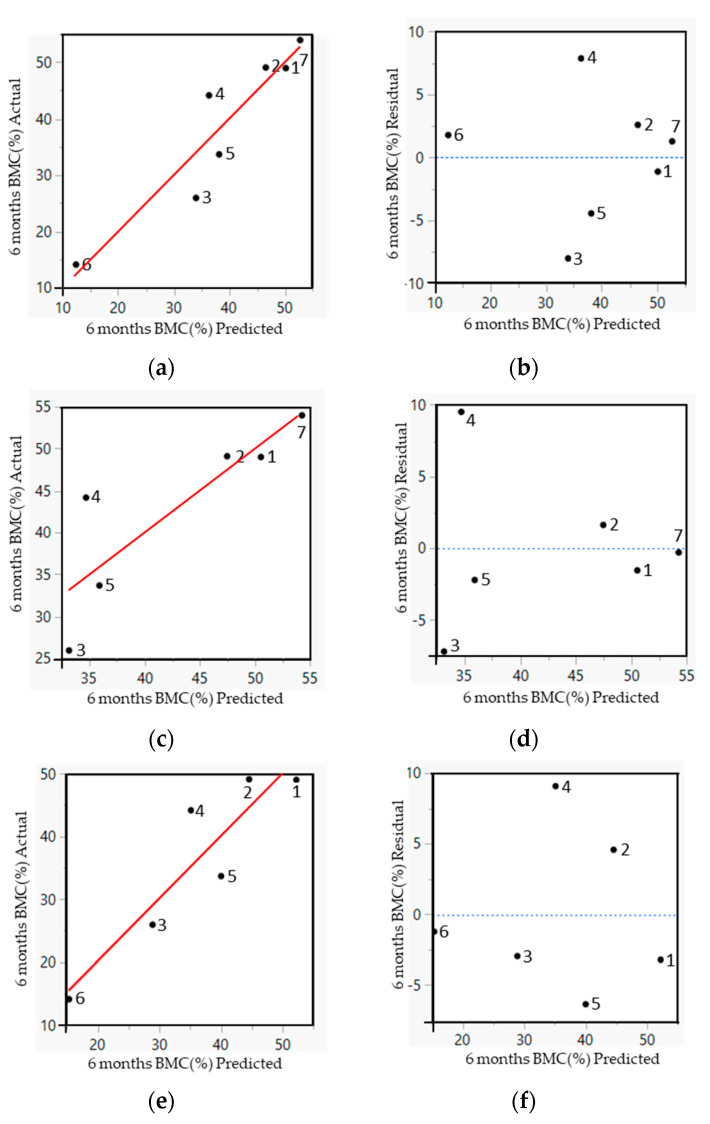
Analysis of the PLSR models. (**a**,**b**) The predicted versus the measured amount of BMC (red line) and the residual by the predicted plot (blue dotted line) for all seven types of biomaterials. (**c**,**d**) The predicted versus the measured amount of BMC (red line) and the residual by the predicted plot (blue dotted line) for six types of biomaterials (all grafts except Natix^®^) indicating their predicted error. (**e**,**f**) The predicted versus the measured amount of BMC (red line) and the residual by the predicted plot (blue dotted line) for six types of biomaterials (all grafts except Ostim^®^) indicating their predicted error. The black dots in the graphs correspond to the following biomaterials: 1-Bio-Oss^®^, 2-Bio-Oss^®^-Collagen, 3-BoneCeramic^®^, 4-Cerasorb^®^, 5-MP3^®^, 6-Natix^®^ and 7-Ostim^®^.

To find out the importance and contribution of physico-chemical characteristics in intra-oral bone regeneration, multivariate statistical analysis using PLSR was implemented to investigate the weighted value of driving biomaterials properties in the bone regeneration process (Figure 5). To achieve the optimized number of dimensions in the PLSR model, the root-mean-squared error (RMSE) with increasing numbers of principal components was calculated for the measured vs. predicted BMC and the minimum RMSE was observed when using two principal components.

In this study, four PLSR models were developed based on introduced factors of the bone grafts along with their *in vivo* response. In the first model, all seven types of bone grafts were included. After a few model iterations, only the weight percentage of CaCO_3_ and Ti along with macroporosity (MP) remained as predictors for the BMC at 6 months after implantation (Equation (4)). Figure 5a,b shows the results of this model.

BMC (%) = 58.14 + 3.6 ∗ CaCO_3_ (wt%) – 0.22 ∗ Ti (wt%) – 0.34 ∗ MP (4)

In order to see the correlation between the physico-chemical properties and the measured amount of BMC in CaP-based bone grafts, a second model was developed, only within this group of biomaterials, excluding Natix^®^. The final PLSR equation predicting BMC at 6 months after implantation contained contributions of the macroporosity and the percentage of CaCO_3_ and H_2_O in the biomaterial (Equation (5)). Figure 5c,d shows the results of this model.

BMC (%) = 49.13 + 4.35 ∗ CaCO_3_ (wt%) + 0.13 ∗ H_2_O (wt%) – 0.29 ∗ MP (5)

In the third model, the only water-containing graft (Ostim^®^) was excluded to see the correlation between the physico-chemical properties and the measured amount of BMC in the absence of H_2_O. The PLSR equation in this model showed the same parameters as the first model indicating the weight percentage of CaCO_3_ and Ti along with macroporosity as determining factors for BMC at 6 months of implantation (Equation (6)). Figure 5e,f shows the results of the third model.

BMC (%) = 93.39 + 3.77 ∗ CaCo_3_ (wt%) – 0.16 ∗ Ti (wt%) – 0.92 ∗ MP (6)

The chemical composition and the macroporosity values in all models were the influencing structural characteristic predicting the contact between the bone and biomaterial. In the first and third models, the same factors were shown to be influencers for the amount of BMC, so excluding the paste-like graft makes little difference to the model. In the second model and by excluding the Ti-containing graft, the weight percentage of Ti was replaced with the H_2_O weight percentage in the model. A substantial correlation (80%) was observed between the predicted and measured amount of BMC, with a low RMSE reducing the risk of overfitting. Having more measures in the equations increased the level of noise and irrelevant information, leading to worse performance. Reducing the amount of measure in the current equations reduced the correlation between the predicted and measured amount of BMC indicating all measures were relevant.

## 4. Discussion

Designing the optimized bone graft for intra-oral applications involves many parameters that directly affect the bone regeneration rate in the defect site. Thus, in order to obtain the optimal scaffold design for a specific application, more insight should be achieved into the influence of biomaterials characteristics on the regeneration process [8]. In this study, we used empirical modeling to assess the weighted value of driving biomaterials properties in the intra-oral bone regeneration process. We used PLSR to construct empirical models that relate combinations of (quantified) biomaterial characteristics to intra-oral bone regeneration outcomes across diverse types of bone biomaterials. This computational method uses linear correlation to reduce the dispersion of a multi-variate data set by identifying the most important information from the original data set. To do so, we fed the models with the topographical (macroporosity and surface roughness) and compositional (chemical components) properties of seven types of commercially available bone grafts as well as their *in vivo* response (bone-to-material contact, being a key parameter for dental applications) when implanted in a sinus defect induced in rabbits. Of these bone grafts, six biomaterials consisted of CaP and only one was made of Ti.

The factors contributing most to the response variable (bone-to-material contact) weighted more heavily in the derived PLSR models. In the first scenario, all seven types of biomaterials, regardless of their composition, were included and the PLSR model showed the importance and influence of chemical composition (CaCO_3_ wt% and Ti wt%) and macroporosity in the healing process of biomaterials. In the second scenario, the only non-CaP-based biomaterial (Natix^®^) was excluded and the PLSR model was developed for the other six tested biomaterials. This model displays again the influence of macroporosity along with the weight percentage of CaCO_3_ and H_2_O in the graft regeneration response. In the third scenario, only the water-containing biomaterial was excluded and the PLSR model exhibited the same drivers as the first model; CaCO_3_ wt%, Ti wt%, and macroporosity. An interesting observation is that excluding the putty graft had a minor impact on the models, demonstrating their robustness.

As observed in Equations (4)–(6), among the non-CaP ingredients of all biomaterials (CaCO_3_, collagen, H_2_O, and Ti), the weight percentage of CaCo_3_ and Ti showed the biggest influence on the BMC values. An interesting aspect of the present models is that CaCo_3_ showed a significant effect on the tissue regeneration responses of the biomaterials despite a much lower amount compared to the other non-CaP ingredients. It should be noted that only Ti and H_2_O became candidates to be excluded from the models as they composed the majority of their own biomaterials.

As reflected in all models, and unsurprisingly, the most effective parameter identified here was the chemical composition. This can be found by the effect of CaCO_3_ and Ti percentages in the first and third models and the effect of water content in the second PLSR model while Natix^®^ was excluded. The effect of chemical composition, particularly for CaP ceramics, has been highlighted previously. Various Ca/P ratios resulting from the diverse chemical compositions lead to different degradation profiles for HAp, TCP, and BCP. A Ca/P ratio of 1.5 for TCP is marked by a high dissolution rate that accelerates material resorption, while pure HAp has a Ca/P ratio of 1.67 and is highly stable [17,18]. Ergun et al. (2007) cultured human osteoblasts on a group of CaPs with Ca/P ratios between 0.5 and 2.5. Results of that study showed that osteoblast adhesion increased on the CaPs with higher Ca/P ratios [19]. The optimization of the chemical phase composition is believed to improve the osteoinductivity and other biological behaviors of CaP ceramics, thereby supporting the restoration of bone defects. Chen et al. (2015) assessed the effect of the chemical phase composition of the porous CaPs and the action mechanism involved by using *in vitro* and *in vivo* evaluations. The results of their *in vitro* cell experiments showed more significant cell proliferation and secretion of angiogenic factors for the CaPs with lower Ca/P ratios compared to the higher ones. Likewise, the *in vivo* assessment in an ectopic implantation model in mice showed more new blood capillaries in the inner pores of the CaPs with lower Ca/P ratios at 2 weeks [20].

Of note though is that the impact of various elements in the models in the current study is different. CaCO_3_ weight percentage in all models showed a significant and positive influence on the contact between bone tissue and biomaterials in the regeneration process; however, the Ti weight percentage, as long as it is included in the models, has a negative impact and in a much lower magnitude. In the second model, the H_2_O weight percentage also showed a lesser but positive impact only when Ti was excluded. Macroporosity was identified as the other key driver for successful bone regeneration. The existence of interconnected macropores was extensively reported as essential for osteogenesis and angiogenesis [21,22,23]. The lowest amount of macroporosity among the biomaterials here was for Ostim^®^ (15.8%) and significantly lower than the average of other biomaterials (63.9%). Therefore, by excluding Ostim^®^ in the third model the impact of macroporosity became stronger in the predicted amount of BMC.

The PLSR analysis used in the current study, using a leave-one-out cross-validation strategy, achieved up to 80% accuracy in predicting the bone forming capacity of bone grafts using five to seven types of biomaterials. Indeed, more samples with a wider range of physico-chemical characteristics would further increase the robustness of the model and reduce the risk of overfitting. For example, including only one non-CaP-based graft in the first and third models did not provide a clear indication of the composition of the optimized graft structure. The percentage of Ti, which was shown as an influencing parameter in those models, varies from 0% for all the CaP-based biomaterials to 100% for Natix^®^. Hence, a second model was created excluding Natix^®^. The biomaterials used in the second model (only CaP-based ones) provided a better range of the relevant material characteristics, with e.g., a water content between 0 and 65% (Ostim^®^). As the macroporosity and chemical composition have been shown to be important drivers in the graft’s performance, the next step is to fabricate the scaffolds with the macroporosity between that of Ostim^®^ and the one of BoneCeramic^®^, and a CaCO_3_ content up to 3.4%. In addition to the aforementioned improvements that can be made to the model, it is important to acknowledge a limitation of the model regarding its ability to accurately reflect the impact of surface roughness on the bone regenerative capacity of scaffolds, despite studies repeatedly demonstrating the importance of surface roughness on the regenerative potential of intra-oral biomaterials [14,15,16]. This could be attributed to the retrospective nature of this study, requiring the use of PMMA-embedded samples to determine the roughness where the accuracy of the roughness profile calculated by the specific MATLAB^®^ tool highly depends on the quality of the ESEM images. This discrepancy suggests that future studies should explore alternative measurement techniques that offer higher sensitivity and quality for imaging of the explants.

In an effort to identify the importance of driving parameters of biomaterials in the tissue healing process, a series of studies have been conducted previously [26,27,28,29]. They have also provided quantitative evidence indicating direct links between biomaterial properties and the tissue formation process. Although they all are dedicated to applications other than intra-oral regeneration, they also identified the importance of both morphological and compositional properties of the scaffold using empirical modeling techniques (multivariate statistics, PLSR). Kerckhofs et al. [26] showed that the pore shape and β-TCP percentage along with the amount of cell seeded were the influencing factors in the bone forming capacity of CaP-based cell-seeded scaffolds after 8 weeks of ectopic *in vivo* implantation. Roberts et al. [27] demonstrated the importance of morphological parameters including surface area, average grain size, and the volume fraction of CaP in the bone formation response of the orthopedic cell-seeded biomaterials after 8 weeks of ectopic *in vivo* implantation. In a similar orthopedic (ectopic) setting, a multivariate statistical analysis was used to gain further insight into the effects of stimulatory factors in skeletal tissue formation after 5 weeks of implantation of cell-based scaffolds coated with recombinant bone morphogenetic protein (BMP)-ligands. That study showed that the type of BMP ligand, as well as the CaP scaffold, affects skeletal tissue formation, observed in both qualitative and quantitative manners (Bolander et al., 2016). In the most recent study in the same ectopic set-up, a design of an experiment approach revealed that cell-seeded CaP scaffolds with an intermediate Ca^2+^ release rate combined with a low or medium dosage of BMP6 demonstrated robust new bone formation after 5 weeks of implantation (Ji and Kerckhofs et al., 2018). In comparison with these studies, the current study is the first, to our knowledge, to correlate several physico-chemical properties and healing capacities of biomaterials investigated in an orthotopic intra-oral bone regeneration setting. Moreover, compared to what has been done in similar studies, neither osteogenic cells nor exogenous biological agents (proteins, growth factors, etc.) were loaded onto the biomaterials in the current study. This enabled us to assess the pure interaction between biomaterials with different topographical and compositional properties and the regeneration potential of the alveolar setting. In the future, more biological players could be drawn into the analysis and the interplay between physico-chemical biomaterial factors and biological ones could be assessed quantitatively for the intra-oral bone biomaterials as well. Nevertheless, the clinical use of cell-based therapies in the dental field might remain limited mainly due to the lack of technological advances and economic reasons, and therefore the morphological features are of major importance [41]. Additionally, the aforementioned studies have developed models with the results of tissue regeneration after a maximum of 8 weeks of *in vivo* ectopic implantation. In the current study, the *in vivo* results used are those obtained after 6 months of orthotopic regeneration. The response of bone regeneration in the longer term can provide a better indication of the actual performance of biomaterials and therefore more robustness in predicting the bone regeneration potential compared to the shorter implantation times.

In terms of providing quantitative insight into the alveolar bone grafts, many meta-analyses also have been conducted in the field of guided bone regeneration [30,31,32]. The focus of these meta-analyses is on the clinical outcome of the different regeneration therapies (e.g., newly regenerated bone, implant survival rate, dimensional changes in the sinus volume, etc.) and not on the influence of specific biomaterial characteristics on these outcomes in alveolar ridge augmentation. Compared to these meta-analyses, the presented study goes a step further to correlate the properties of implanted biomaterials to bone regeneration outcomes.

Another computer modeling approach used in the design of optimal biomaterials for bone regeneration purposes is based on the mechanistic principles of tissue formation as investigated in the field of curvature biology [42,43,44,45]. Indeed, this concept links the tissue growth dynamics to the fundamental interactions between cells and certain morphological factors of the substrate. For example, Gamsjäger et al. presented a theoretical framework linking tissue growth to the mechanotransduction pathways (in particular surface stress and strain) activated when the cells are attached to biomaterial substrates having a particular pore curvature (Gamsjäger et al., 2013). These curvature growth-based models provide a mechanistic basis for biomaterial optimization. Recent extensions to these models would allow the incorporation of the effects of other factors (such as oxygen or growth factors) on the tissue growth dynamics [46,47]. Such mechanistic models can be used either as a stand-alone tool to optimize biomaterials (as demonstrated by the authors in [48]), or they can be used to identify crucial material characteristics that can be added next to the mechanistic models and contribute to the design of optimal biomaterials that way.

As mentioned before, despite the relatively large set of empirical data available on bone graft characteristics, there is still a need for a quantitative understanding of their importance and contribution to the bone regeneration process. There are many screening studies about alveolar bone grafts [10,14,24,33,34,35], but the reporting of data is often inconsistent or insufficiently documented, and few studies focus on the influence of specific combinations of physico-chemical characteristics on intra-oral bone regeneration. Furthermore, the large range of characteristics for specific biomaterials resulting from variations in the fabrication methods can lead to unpredictable outcomes in the bone regeneration process. The optimal bone graft is still an unmet need, requiring accuracy, robustness, and mechanistic insight to facilitate the design of the next generation of bone grafts. This may be facilitated by using computational (empirical and/or mechanistic) modeling to identify the required material characteristics and the use of new production technologies to manufacture them. Additive manufacturing technologies such as three-dimensional (3D) printing provide the ability to create bone scaffolds with controlled chemistry, topography, shape, and porosity as well as personalized bone grafts for tailored patient-specific and defect-specific clinical conditions.

## 5. Conclusions

In conclusion, the presented model provides a first step in the identification of biomaterial properties and morphological cues driving the intra-oral bone healing process as well as predict the bone regeneration potential of new biomaterials based on several physico-chemical characteristics. This tool can be used for the rational design of (3D printable) bone biomaterials with a more controlled and custom-made structure, ultimately facilitating and improving clinical translation.

## Figures and Tables

**Table 1:** The chemical composition of different alveolar grafts along with their origin, physical form, and particle size provided by the manufacturers. Images Copyright © by the distributors for images in the ‘Figure’ column. Right column contains scanning electron microscopy (SEM) image of grafts 1 week after implantation (scale bar: 1 mm) [33,34,35].

Trade Name	Chemical Composition (wt%)	Origin	Physical Form	Particle Size (µm)	Figure	SEM Micrograph
Bio-Oss^®^	93.6%HAp + 3.4%CaCo3 + 3%COL	Bovine	Solid granulates	250–1000	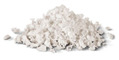	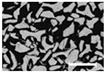
Bio-Oss^®^-Collagen	Bio-Oss^®^ + 10%COL	Bovine/Porcine	Solid granulates in a collagen matrix	250–1000	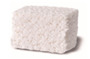	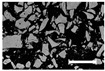
MP3^®^	90%bone mix + 10%COL	Porcine	Pre-hydrated granulates in a collagen matrix	600–1000	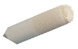	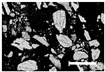
Ostim^®^	35%HAp + 65%H2O	Synthetic	Granular paste	0.001–0.05	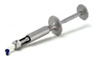	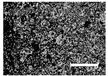
Cerasorb^®^	100%β-TCP	Synthetic	Solid granulates	500–1000	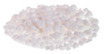	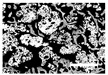
BoneCeramic^®^	60%HAp + 40%β-TCP	Synthetic	Solid granulates	400–700	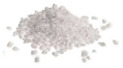	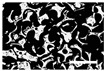
Natix^®^	100%Ti	Synthetic	Solid granulates	700–1000	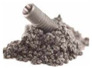	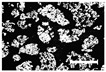

## Data Availability

The data presented in this study are available on request from the corresponding author. The data will be available in a publicly accessible repository.
